# Can X-Ray Powder Diffraction Be a Suitable Forensic Method for Illicit Drug Identification?

**DOI:** 10.3389/fchem.2020.00499

**Published:** 2020-06-23

**Authors:** Bronislav Jurásek, Vilém Bartůněk, Štěpán Huber, Patrik Fagan, Vladimír Setnička, František Králík, Wim Dehaen, Daniel Svozil, Martin Kuchař

**Affiliations:** ^1^Forensic Laboratory of Biologically Active Substances, Department of Chemistry of Natural Compounds, University of Chemistry and Technology Prague, Prague, Czechia; ^2^Department of Inorganic Chemistry, University of Chemistry and Technology Prague, Prague, Czechia; ^3^Department of Analytical Chemistry, University of Chemistry and Technology Prague, Prague, Czechia; ^4^CZ-OPENSCREEN: National Infrastructure for Chemical Biology, Department of Informatics and Chemistry, Faculty of Chemical Technology, University of Chemistry and Technology Prague, Prague, Czechia

**Keywords:** new psychoactive substances, X-ray powder diffraction, drug detection, infrared spectroscopy, Raman spectroscopy

## Abstract

New psychoactive substances (NPSs) are associated with a significant number of intoxications. With the number of readily available forms of these drugs rising every year, there are even risks for the general public. Consequently, there is a high demand for methods sufficiently sensitive to detect NPSs in samples found at the crime scene. Infrared (IR) and Raman spectroscopies are commonly used for such detection, but they have limitations; for example, fluorescence in Raman can overlay the signal and when the sample is a mixture sometimes neither Raman nor IR is able to identify the compounds. Here, we investigate the potential of X-ray powder diffraction (XRPD) to analyse samples seized on the black market. A series of psychoactive substances (heroin, cocaine, mephedrone, ephylone, butylone, JWH-073, and naphyrone) was measured. Comparison of their diffraction patterns with those of the respective standards showed that XRPD was able to identify each of the substances. The same samples were analyzed using IR and Raman, which in both cases were not able to detect the compounds in all of the samples. These results suggest that XRPD could be a valuable addition to the range of forensic tools used to detect these compounds in illicit drug samples.

## Introduction

The pharmacophore is the part of the chemical structure that is responsible for the biological effect of the substance. Thus, if the structure of a chemical entity is modified without affecting the pharmacophore, this substance will very likely retain the biological effects of the starting compound. These findings are widely used in drug design; however, the pharmacophore theory has also begun to be used in the illicit drug scene over the last decade. If the structure of an illicit drug is modified while retaining its pharmacophore, the newly prepared entity will not be covered by the current legislation, while its effects will very likely be similar to the already banned unaltered substance. These substances [called new psychoactive substances (NPSs) or designer drugs] are being monitored by the European Monitoring Centre for Drugs and Drug Addiction (EMCDDA). The latest EMCDDA annual drug market report mentions about 730 different NPSs. Although the annual growth of these substances has decreased over the last 2 years (about 1 new substance per week), according to the latest EMCDDA report there are ~400 NPSs appearing on the market regardless of any regulations (European Monitoring Centre for Drugs Drug Addiction, 2018; European Monitoring Centre for Drugs Drug Addiction Europol, 2019). The increase of both substances that are completely new on the market and those that occur on the market regardless of any legal regulations already exerts considerable pressure on the analytical teams monitoring these compounds. Moreover, psychoactive substances are often sold as blends, which complicates their detection further. Hence, there is a significant demand for the development of easy, fast and reliable field detection methods for psychoactive substances (European Monitoring Centre for Drugs Drug Addiction, 2018; European Monitoring Centre for Drugs Drug Addiction Europol, 2019).

United Nations Office on Drugs and Crime (UNODC) has published (UNODC - United Nations Office on Drugs Crime, 2013) a study that has gathered data on the methods used for the identification of NPSs from 60 countries (Popovic et al., 2019). According to the respondents, the mostly used group of methods belong to the chemical analysis techniques [i.e., gas chromatography—mass spectrometry (GC-MS), liquid chromatography—mass spectrometry (LC-MS), high performance liquid chromatography (HPLC), vibrational spectroscopies—Fourier transform infrared spectroscopy (FTIR) and Raman spectroscopy, and nuclear magnetic resonance (NMR)]. While GC and LC enable separation of the analytes and thus may provide both qualitative and quantitative analysis, NMR is especially valuable due to its potential to elucidate unknown structures in the samples. However, all of these instrumentations have to be used wisely, with regard to their individual strengths and limitations (complex mixtures renders the NMR and FTIR spectra too complex, while isomers may complicate the use of GC-MS or LC-MS techniques; UNODC - United Nations Office on Drugs Crime, 2013).

The choice of analytical instrumentation is often limited by the type of the sample. Biological samples such as blood (Mercieca et al., 2018), hair (Kyriakou et al., 2017; Salomone et al., 2017; Fabresse et al., 2019), or urine (Meyer et al., 2016; Vikingsson et al., 2017; Mercieca et al., 2018) are a relatively complex matrix for analysis. Therefore, considerable effort has been invested into development of separation methods coupled with mass detection, which are, together with immunochemical methods (Cannaert et al., 2018; Maryška et al., 2018), currently the main techniques for NPS identification in biological matrices. Furthermore, the sensitivity of current techniques and the knowledge of psychoactive substance metabolisms (Vikingsson et al., 2015, 2017; Šícho et al., 2019) allow their detection also in local wastewater (González-Mariño et al., 2013; Rosi-Marshall et al., 2015; Croft et al., 2020) (e.g., festivals, prisons) and thus offer valuable data on the prevalence (Mastroianni et al., 2016; Croft et al., 2020) of individual substances in society.

Substances of certain groups (e.g., cannabinoids) are often distributed to the end user applied on another medium, such as dried herbal leaves (Ciolino, 2015; Namera et al., 2015) (e.g., damiana) or on paper and, as such, smuggled into prisons in letters or books (Metternich et al., 2019; Hvozdovich et al., 2020). Although after appropriate sample treatment a wide selection of techniques can be used for the analysis, LC-MS seems to be the most prevalent one (UNODC - United Nations Office on Drugs Crime, 2013). Mass spectrometry seems to be also used to analyse seized psychoactive substances in its powder form even though such samples can be analyzed by any of the aforementioned methods. Although a tandem of separation technique with a mass detector appears to be the universal method (Pasin et al., 2017), it requires an experienced operator for its use and maintenance, on top of the often required standards. This renders the analyses expensive (Pasin et al., 2017). Therefore, from the perspective of price efficiency, there is still a significant demand for less resource intensive yet reliable analytical alternatives.

Infrared (IR) and Raman spectroscopies belong to the other most common choices for the analysis of solid illicit street drug samples as they generally enable a fast and relatively cheap analysis (Stewart et al., 2012; Jones et al., 2016; Maheux et al., 2016; Apirakkan et al., 2018; Pereira et al., 2018). Their application does not demand a complicated sample preparation and commercially available portable spectrometers offer the possibility of *in situ* measurements (Correia et al., 2018; Yu et al., 2018). However, these methods also have some limitations. In case of Raman spectroscopy, a high level of fluorescence caused either by an active substance or by an additive may complicate the interpretation of the spectra. Furthermore, in the case of complex mixtures (e.g., heroin street samples generally do not contain more than 30% of the active substance) (Fabresse et al., 2019), the interpretation of the IR and Raman spectra may be very difficult due to interfering bands of various adulterants.

Hence, we investigated the potential of X-ray powder diffraction (XRPD) in the analysis of solid samples seized on the black market. XRPD, a widely used analytical technique in the pharmaceutical industry, has been used in several forensic cases involving analyses of soils (Kotrlý, 2006), where it offered fruitful results for the investigation, so it is an already established analytical technique in forensic sciences (Thatcher and Briner, 1986). Although it has a relatively wide possibilities of its use in forensics, including analyses of explosives (Thatcher and Briner, 1986; Kotrlý, 2006), fibers (Thatcher and Briner, 1986) or illicit drugs including some of the cutting agents (Folen, 1975; Thatcher and Briner, 1986), its use has been rather neglected in this field. Moreover, the situation in the field of psychoactive substances has changed dramatically with the NPSs entering the drug market in recent years. XRPD represents a simple, non-destructive technique enabling the reliable identification of either pure solid substances or their street sample mixtures. Moreover, it might also be able to distinguish inorganic compounds (e.g., gypsum) that might be often life threatening when injected. XRPD may serve as a suitable complementary method to vibrational spectroscopy for the analysis of various seized street drug samples that may especially help in cases where fluorescence or the varied composition of the analyzed samples hinder the routine identification by Raman or IR spectroscopies. However, the scope of XRPD is limited solely to use on solid samples. In our previous work we have shown on a series of cathinones that this method can not only distinguish between structurally similar NPSs, but that it can also identify substances in mixtures (Jurásek et al., 2019). In this work, we analyzed cocaine, heroin, and 5 NPS street samples with their respective standards by XRPD and the results were compared with the commonly used IR and Raman spectroscopy measurements.

## Experimental Section

### Analyzed Samples

The origin and specifications of all the analyzed samples are given in Table 1. The real samples, which were collected with standard ethical procedure, were provided as a part of the seizures performed by the Police of the Czech Republic, while the standards (purity of all the used standards were higher than 98%) were acquired from different sources (Table 1). Samples 5F-ADB I. and II. were provided as a part of seizures performed by the Police of the Czech Republic, while the samples 5F-ADB IV., V., and VI. were obtained from a commercial vendor in the framework of a darknet study. Sample 5F-ADB III. was prepared in house.

**Table 1:** Overview of the tested samples and standards.

**Sample name**	**Chemical name**	**Structure**	**Origin**
Heroin seized	(5α,6α)-7,8-didehydro-4,5-epoxy-17-methylmorphinan-3,6-diol diacetate	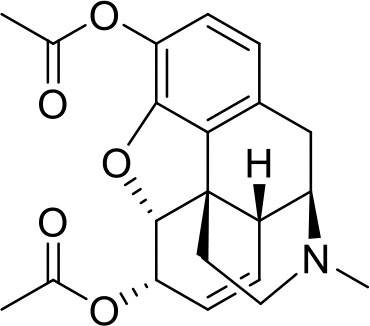	Sample was seized by the Police of the Czech Republic.
Heroin standard			Standard was purified in our laboratory.
Cocaine seize	methyl (1S,3S,4R,5R)-3-benzoyloxy-8-methyl-8-azabicyclo[3.2.1]octane-4-carboxylate	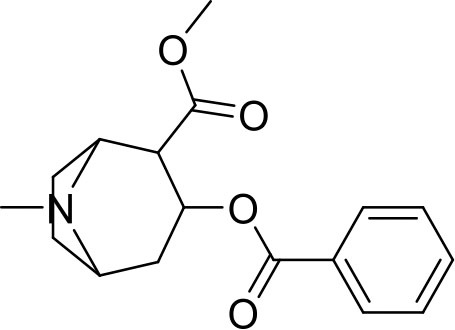	Sample was seized by Police of the Czech Republic.
Cocaine standard			Standard purchased from Fagron a.s.
Mephedrone seized	2-(methylamino)-1-(4-methylphenyl)propan-1-one	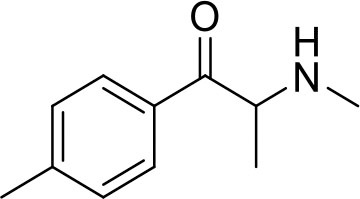	Sample was seized by Police of the Czech Republic.
Mephedrone standard			Standard was purchased from Alfarma s.r.o.
Ephylone seized	1-(1,3-Benzodioxol-5-yl)-2-(ethylamino)pentan-1-one	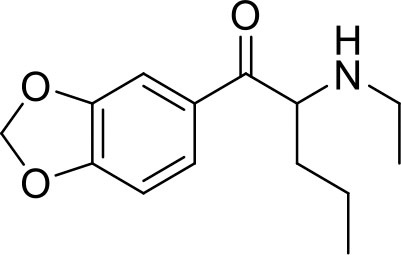	Sample was seized by Police of the Czech Republic.
Ephylone standard			Sample was purified in our laboratory.
Butylone seized	1-(1,3-benzodioxol-5-yl)-2-(methylamino)butan-1-one	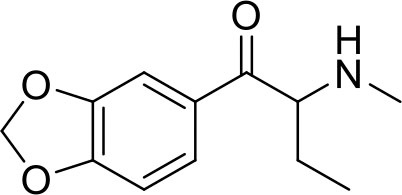	Sample was seized by Police of the Czech Republic.
Butylone standard			Standard was purchased from Alfarma s.r.o.
JWH-073 seized	Naphthalen-1-yl-(1-butylindol-3-yl)methanone	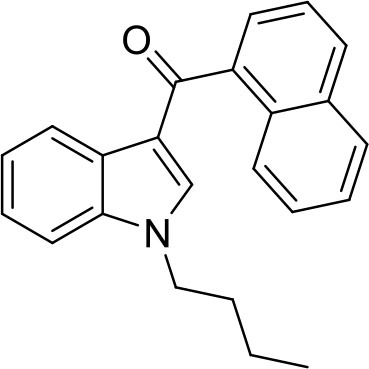	Sample was seized by Police of the Czech Republic.
JWH-073 standard			Standard was purchased from Alfarma s.r.o.
2-naphyrone seized	1-(naphthalen-2-yl)-2-(pyrrolidin-1-yl)pentan-1-one	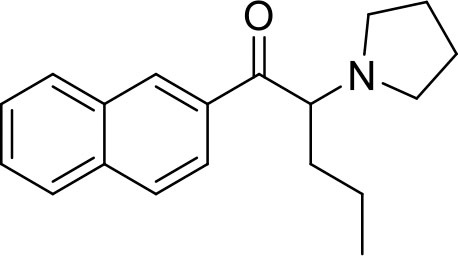	Samples were seized by Police of the Czech Republic.
2-naphyrone standard			Standard was purchased from Alfarma s.r.o.

### X-Ray Powder Diffraction

The sample crystals were crushed with a microscope slide on a silicon pod (see Supplementary Figure 1) and, thus, a narrow surface was created. For the remeasurement, JWH-073 samples were ground extensively in the agate mortar to show the differences in relative intensities.

The XRPD data were collected with a Bruker 2nd generation D2 Phaser powder diffractometer (Bruker AXS, Germany) at room temperature with parafocusing Bragg-Brentano geometry using CuKα radiation (λ = 1.5418 Å, U = 30 kV, I = 10 mA). The data were scanned with an ultrafast LYNXEYE XE detector over the angular range of 5 to 60 °2θ with a step size of 0.019 °2θ and a counting time of 1 s per step. The software HIGHSCORE PLUS 3.0e (PANalytical, Almelo, Netherlands) was employed to fit the background using a polynomial method, to smooth the data and to eliminate the Kα2 component. The top of the smoothed peaks was used to determine the peak positions and intensities. Determination of peak positions was made by an in-build algorithm in the HIGHSCORE PLUS 3.0e. Thus, processed diffraction patterns were subjected to the pattern searching procedure within PDF4+ database via the HighScore software.

To compare similarity of XRPD diffractograms quantitatively, a cross-correlation score was used. To accentuate peaks and attenuate background noise and minor pollutant effects, we pre-prepare the normalized patterns by squaring them (Equations 1, 2) and only then calculating their cross-correlation (Equations 3, 4). With f'(θ) as the normalized XRPD diffractogram of a known standard and g'(θ) as the normalized XRPD diffractogram of a measured sample, we calculate a cross-correlation score CCS_fg_ as defined below(Equation 5):

(1) $$\text{Squared XPRD pattern of standard}:\text{ f}(\theta )={\text{f}}^{\prime 2}(\theta )$$

(2) $$\text{Squared XPRD pattern of sample}:\text{ g}(\theta )={\text{g}}^{\prime 2}(\theta )$$

(3) $$\text{Cross-correlation}:{\text{C}}_{fg}(\tau )≜\int_{-\infty }^{\infty }\bar{f(\theta )}g(\theta +\tau )d\theta$$

(4) $$\text{Auto-correlation}:{\text{C}}_{ff}(\tau )≜\int_{-\infty }^{\infty }\bar{f(\theta )}f(\theta +\tau )d\theta$$

(5) $$\begin{array}{l} \text{Cross}-\text{correlation score: CC}{\text{S}}_{fg}≜\frac{\overset{\infty }{\underset{-\infty }{\int }}C_{fg}\left(\tau \right)d\tau }{\overset{\infty }{\underset{-\infty }{\int }}C_{ff}\left(\tau \right)d\tau } \end{array}$$

A discrete calculation method for CCS_fg_, directly using ASC files, was implemented as a Python script (available at https://github.com/dehaenw/cross-correlation).

### Optical Microscopy

Crystal shapes were visualized by confocal microscope Olympus Lext OLS 3100 without any additional image processing.

### Infrared and Raman Spectroscopy

The IR spectra of the standards and real samples were measured on a FT-IR Nicolet iS50 spectrometer (Thermo Scientific, USA) with a Tungsten-halogen MIR radiation source, KBr beam splitter and DLaTGS detector. All the samples were in the form of a powder and they were analyzed by the ATR technique with a diamond crystal. The spectra were recorded in a spectral region of 4,000–400 cm^−1^ with a resolution of 4 cm^−1^ and they are presented as an average of 256 scans. The spectral background was collected before every sample measurement.

The Raman spectra were acquired on a DXR SmartRaman spectrometer (Thermo Scientific, USA) equipped with two lasers (excitation wavelengths 532 and 780 nm). In the case of the 532 nm laser, a diffraction grid comprised of 900 lines per mm, a laser power of 5 mW and 10 accumulations each of 10 s exposure time were used. A diffraction grid with 400 lines per mm, a laser power of 65 mW and 10 accumulations each of 10 s exposure time were used for the measurements with the 780 nm laser. The spectra were recorded in a spectral region of 400–3,000 cm^−1^ with a resolution of 2.4–4.4 cm^−1^ and all the samples were analyzed in glass vials. The spectra were processed with the correction of fluorescence (6th order polynomial).

## Results and Discussion

### X-Ray Powder Diffraction

**Figure 1:**
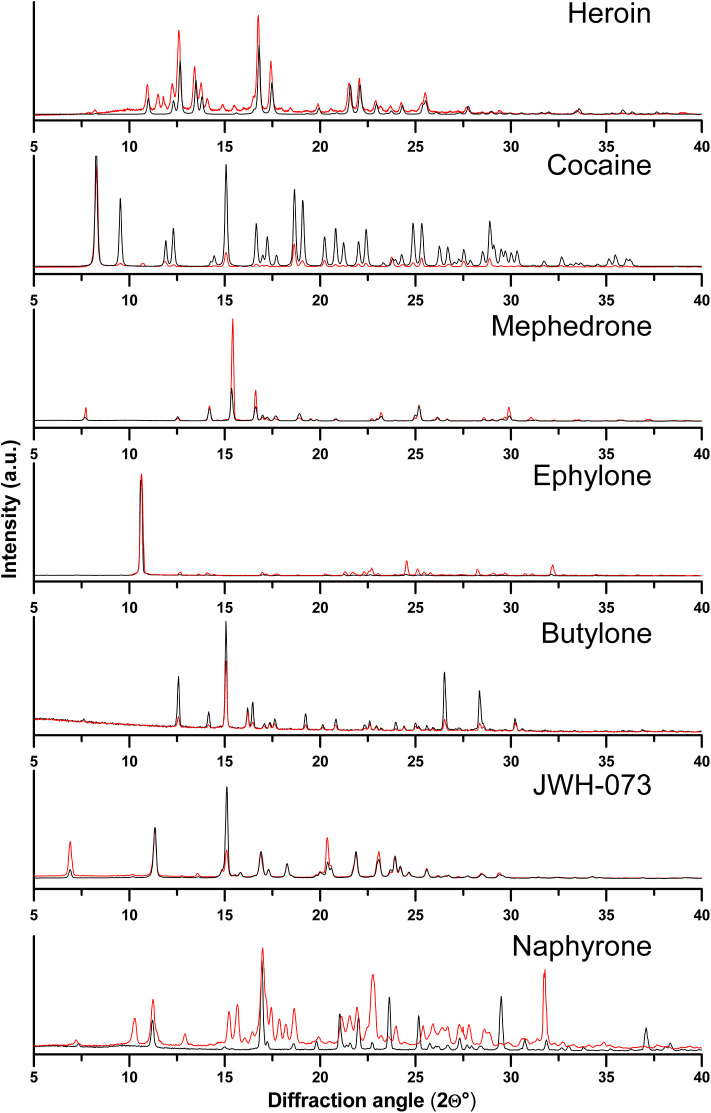
Diffraction patterns of heroin, cocaine, mephedrone, ephylone, butylone, JWH-073, and naphyrone samples. The red line marks the seized samples and the black marks the standards.

We have demonstrated in our previous work (Jurásek et al., 2019) that XRPD can effectively distinguish between particular chemical entities even with similar chemical structures, which is essential in the forensic analysis of NPSs. However, most of the tested standards were prepared in our laboratory and the results were not extensively compared with authentic samples that might occur on the drug market (Jurásek et al., 2019). Therefore, in the current study, we analyzed 7 samples of psychoactive substances (cocaine, heroin, and 5 NPSs) that were seized on the black market and the acquired results were compared with the diffraction patterns of the respective standards (Figure 1).

PDF4+ is a commercially available database that contains more than 410 000 diffraction patterns. Although this database does not contain most NPSs, this database contains diffraction patterns of heroin and cocaine. Such a big amount of patterns in this database made us wonder if it would be possible to use it for identification of street samples of heroin and cocaine. The results were quite impressive, as we were able to identify both cocaine and heroin in street mixtures (see non-modified search data in the Supplementary Data) using this commercial database. However, we were unable to assign the cutting agents, as this database mostly does not contain their respective patterns. Because there is no suitable database of illicit substances yet, street samples of NPSs were just compared with the diffraction patterns of their respective standards.

**Figure 2:**
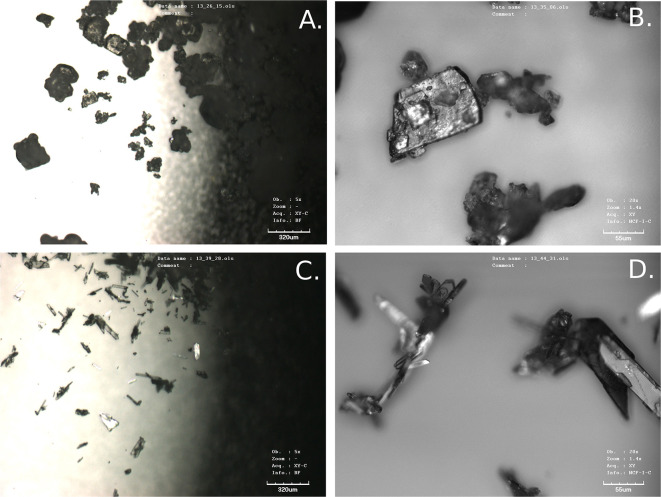
Confocal microscopy visualizations of JWH-073 standard (**A,B**—closer detail) and seized sample (**C,D**—closer detail).

**Figure 3:**
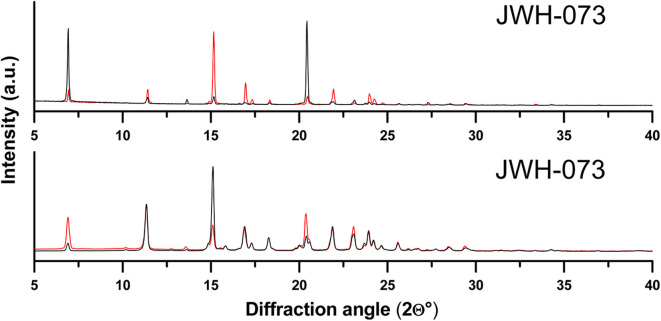
Diffraction patterns of JWH-073 sample and the respective standard remeasured after grinding in the agate mortar.

JWH-073 was identified by XRPD (Figure 1) in one of the seized materials despite the observation that relative intensities in the diffraction pattern differed considerably. The most intensive peak of the standard sample was 15.1 °2θ whereas in the seized sample it was 21.5 °2θ. However, these differences in the relative intensities might be caused by different crystal shapes. To confirm that the standard and seized sample had different crystal shapes, they were subjected to a visual analysis by the optical confocal microscope (Figure 2). The crystal proportions of the seized sample were approximately the same in all three dimensions, but the standard formed needle-like shapes and so one dimension was significantly larger than the other two. This was presumably caused either by the type of crystallization or the synthetic process of the respective samples (Morris et al., 2000). Therefore, to reduce the differences in relative intensities both JWH-073 sample and the respective standard were extensively ground in an agate mortar and remeasured with the same setting of the goniometer (see Figure 3). The differences in the signal intensities did not have any effect regarding the identification of the compound in the seized sample as the peak positions did not change. The sample was successfully identified according to the peak positions and no other peaks were observed suggesting a high purity of the JWH-073 in the seized material.

Mephedrone, ephylone, naphyrone, and butylone were successfully identified in the seized material by comparison of the seized samples and respective standard diffraction patterns. Although the relative intensities of some peaks differed slightly in both samples, which was presumably caused again by different crystal shapes, there were no other peaks at different positions suggesting that the seized materials were of high purity.

Although cocaine and heroin do not belong to the NPSs, the prevalence of these compounds on the drug market is high and therefore we have included these “classic” street drugs in the examination test of XRPD in a similar manner as in the case of the NPSs. Since cocaine and heroin have been already measured and their diffractograms were included in the database of PDFs (powder diffraction file), database cards were used for the identification instead of using the respective standards.

This approach was chosen mainly to prove that the samples could be identified without the need of a standard only by using a suitable database. The seized sample of heroin was successfully identified as diacetylmorphine with card PDF 00-033-1635 when most of the peaks belonged to the drug. The relatively intensive peaks 11.5 and 11.8 °2θ might be attributed to possible diluting agents (e.g., caffeine, PDF 00-049-2058). However, the aim of this study was to prove that XRPD can be used for drug identification and therefore, these impurities were not further investigated. Cocaine was identified by XRPD in the last seized sample. All of the major peaks were attributed to cocaine hydrochloride (PDF 00-030-1629) with the exception of the less intensive peak 10.7 °2θ and a few minor peaks, which might be attributed to specific adulterants in the future.

### Comparison With Vibrational Spectroscopy Measurements

To compare the efficiency of XRPD with other non-destructive methods that are often used in forensic practice, all of the samples and standards were measured by the IR and Raman spectroscopies. The differences of the results provided by these methods have been highlighted.

**Figure 4:**
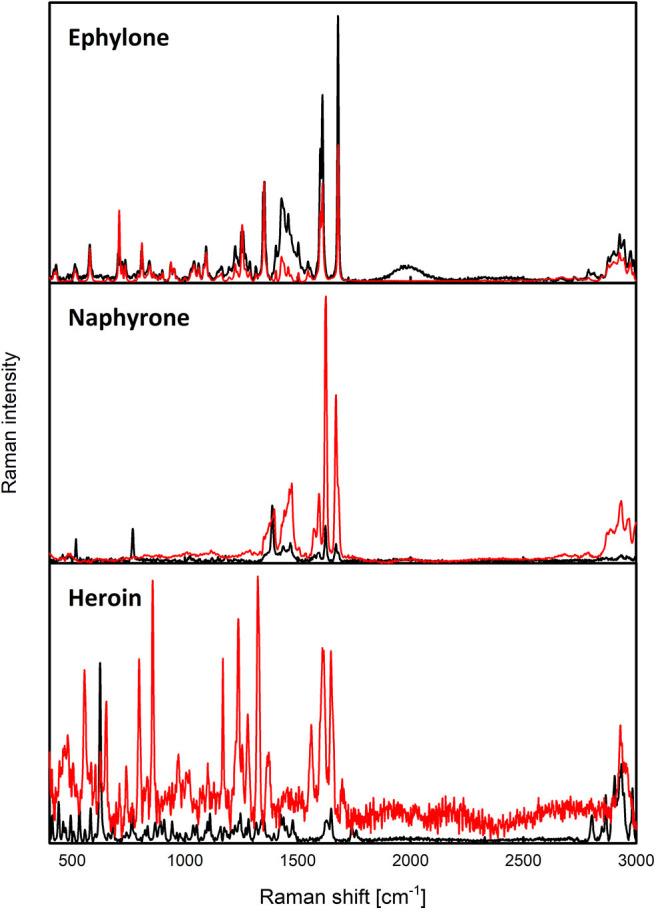
Raman spectra of ephylone, naphyrone, and heroin. The red line marks the seized samples and the black marks the standards.

The Raman spectroscopy suffered from the high fluorescence level with the use of the 532 nm excitation wavelength, where only measurements of the real sample of JWH-073 provided an interpretable spectrum. After the application of the 780 nm excitation wavelength, the fluorescence level decreased in most cases and the active substances were identified by a simple comparison with the spectra of the corresponding standards (Figure 4 and Supplementary Data). However, the high level of fluorescence made the analysis of the naphyrone street sample impossible (Supplementary Data) and the high amount of the adulterants in the heroin sample did not allow a reliable identification of the active substance (Figure 4).

**Figure 5:**
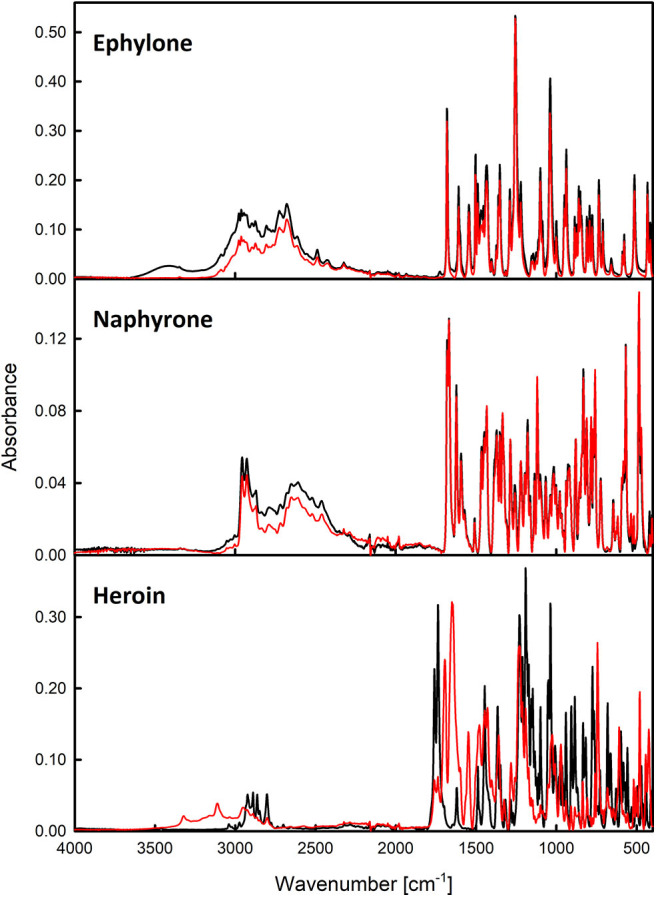
IR spectra of ephylone, naphyrone, and heroin. The red line marks the seized samples and the black marks the standards.

The IR spectroscopy performed slightly better, as it allowed the reliable identification of 6 of the 7 analyzed samples (Figure 5 and Supplementary Data), but the identification of the active substance in the heroin street sample was not possible due to the presence of many interfering bands. Both IR and Raman spectroscopies can offer spectra within several minutes, whereas XRPD instrumentation is usually more time demanding (about 15–20 min). All the measured data can be found in the Supplementary Data.

Vibrational spectroscopy proved to be a powerful tool in the analysis of illicit drug samples as was expected. However, in the case of the heroin sample, both methods struggled in the identification of the active substance. On the other hand, heroin was easily identified by XRPD, we thus believe that its potential in the forensic practice is promising.

**Figure 6:**
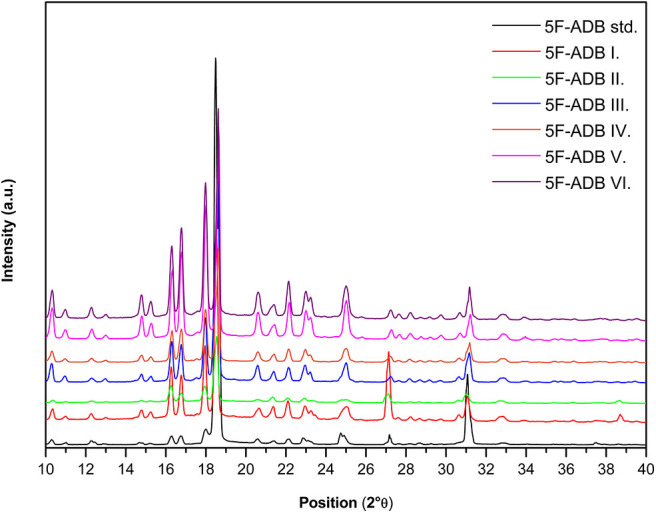
Diffraction patterns of 5F-ADB samples. The color lines marks the seized samples and the black marks the standards.

Although differences in relative intensities in the XRPD patterns may seem to complicate the identification of unknown substances, on the contrary, in some cases, it might further provide valuable data about the analyte (e.g., differences between the production procedures). To demonstrate this ability, six different samples of the 5F-ADB, which were obtained during our NPS survey in the Czech Republic, were analyzed and compared with the standard. The same peak positions in the diffraction patterns enabled the identification of the 5F-ADB in the samples. Two of the samples offered diffraction patterns with not only the same peak position but the relative intensities corresponded as well. The other samples exhibited differences in relative intensities (see Figure 6).

**Figure 7:**
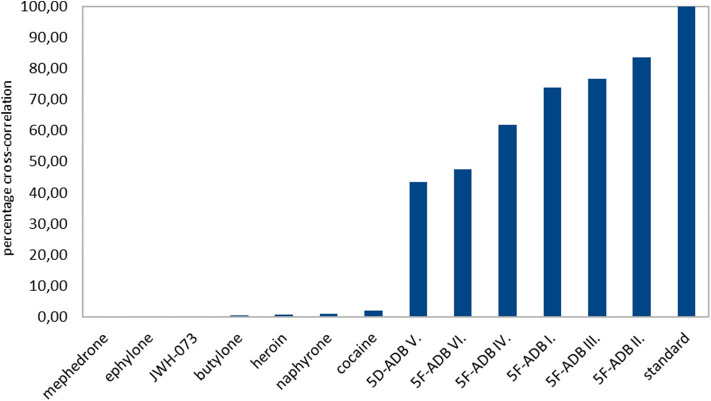
A cross-correlation score for the quantitative comparison of XRPD diffractograms.

This difference in relative intensities makes qualitative estimation of similarity by visual comparing and simple pattern subtracting suggestive but tricky and non-trivial. As a quantitative similarity approach, we proposed a cross-correlation score. This showed that the XRPD patterns of all seven different samples of 5F-ADB have a much higher score than the diffractograms of other, unrelated compounds (see Figure 7).

This suggests that peak positions are essential for the substance identification, whereas an exact match of the relative intensities is not needed, as is a well-known property of XRPD patterns (Pecharsky and Zavalij, 2009). A match of the relative intensities occur when the shape of the crystals and partly their size correspond. Interestingly, we observed a similar relative intensity pattern for sample V. and VI., which could be possibly attributed to their shared origin. If so, such information may be useful for investigators as samples with the same relative intensities could either be from the same source or possibly even from the same batch. Yet, drawing such conclusions based on the agreement of relative intensities may be unreliable, therefore the use of LC-MS remains the only reliable and generally powerful method for this purpose. In case a reliable correlation could be found generally, XRPD may be useful as a pre-screening method for this purpose. Notably, significant grinding of the samples undoubtedly affects the relative intensity pattern of the measured samples (see Figure 3), which unfortunately further complicates the option to compare “similarity of the samples” in a straightforward manner.

Nevertheless, it is essential to note that if it is not possible to assign all the signals in the diffraction pattern, then further analyses may be required. However, after creating a robust database of diffraction patterns of NPSs and cutting agents, such database would enable the identification of not only the main compound but also help with assigning all of the other signals in the pattern to other compounds.

## Conclusions

Seized samples of heroin, cocaine and 5 NPSs (mephedrone, ephylone, butylone, JWH-073, and 2-naphyrone) were analyzed by XRPD, IR, and Raman spectroscopies and compared to the standards of the respective substances. We have shown that XRPD detected all of the analyzed NPSs, as well as providing a reliable identification of the “traditional” drugs cocaine and heroin. In the latter case, the methods of vibrational spectroscopy struggled with the identification of the active substance, while XRPD provided a convincing result, which documents its promising potential in the field of the forensic practice. This instrumentation is not omnipotent, (nor are any other instrumentation currently being used in forensics). However, further combination of XRPD with vibrational spectroscopic methods can effectively eliminate the shortcomings of each of the methods and thus increase the overall reliability of the analysis. Moreover, we believe that in the future, when an appropriate database becomes available, this technique will have the potential to become a strong forensic tool.

## Data Availability Statement

The datasets generated for this study are available on request to the corresponding author.

## Author Contributions

BJ and MK designed the experiment. FK measured the IR spectra and PF measured the Raman spectra. BJ, VB, and ŠH measured the XRPD. ŠH, VS, and PF evaluated the XRPD, IR, and Raman data. WD and DS developed, tested and applied Python script for cross-correlation calculations. BJ, VB, and FK prepared the manuscript. MK, VS, and DS proofread the manuscript. DS, BJ, and VB revised the manuscript.

## Conflict of Interest

The authors declare that the research was conducted in the absence of any commercial or financial relationships that could be construed as a potential conflict of interest.
