# Supplementary material for: Can X-Ray Powder Diffraction Be a Suitable Forensic Method for Illicit Drug Identification?
Source: Front Chem. 2020 Jun 23;8:499. doi: 10.3389/fchem.2020.00499 (PMC7325198; doi:10.3389/fchem.2020.00499)
Supplement: Supplementary file 1 [file Data_Sheet_1.docx]

Supplementary Material

# Contents

[1 Contents 1](#_Toc39574138)

[2 Cocaine 4](#_Toc39574139)

[3 Heroin 9](#_Toc39574141)

[4 Mephedrone 13](#_Toc39574142)

[5 Ephylone 16](#_Toc39574143)

[6 Butylone 19](#_Toc39574145)

[7 JWH-073 22](#_Toc39574146)

[8 Naphyrone 25](#_Toc39574147)


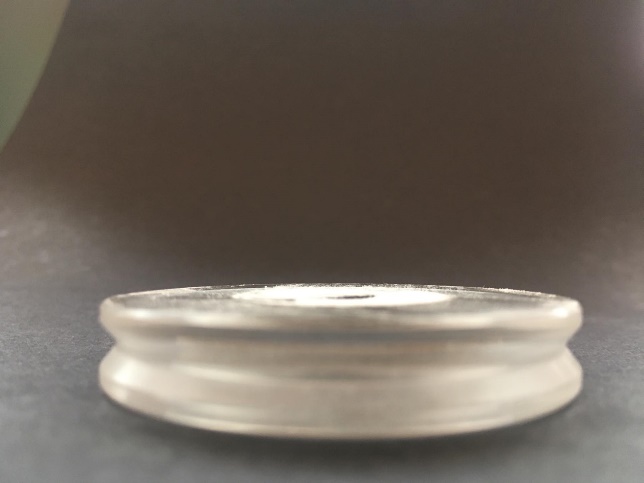

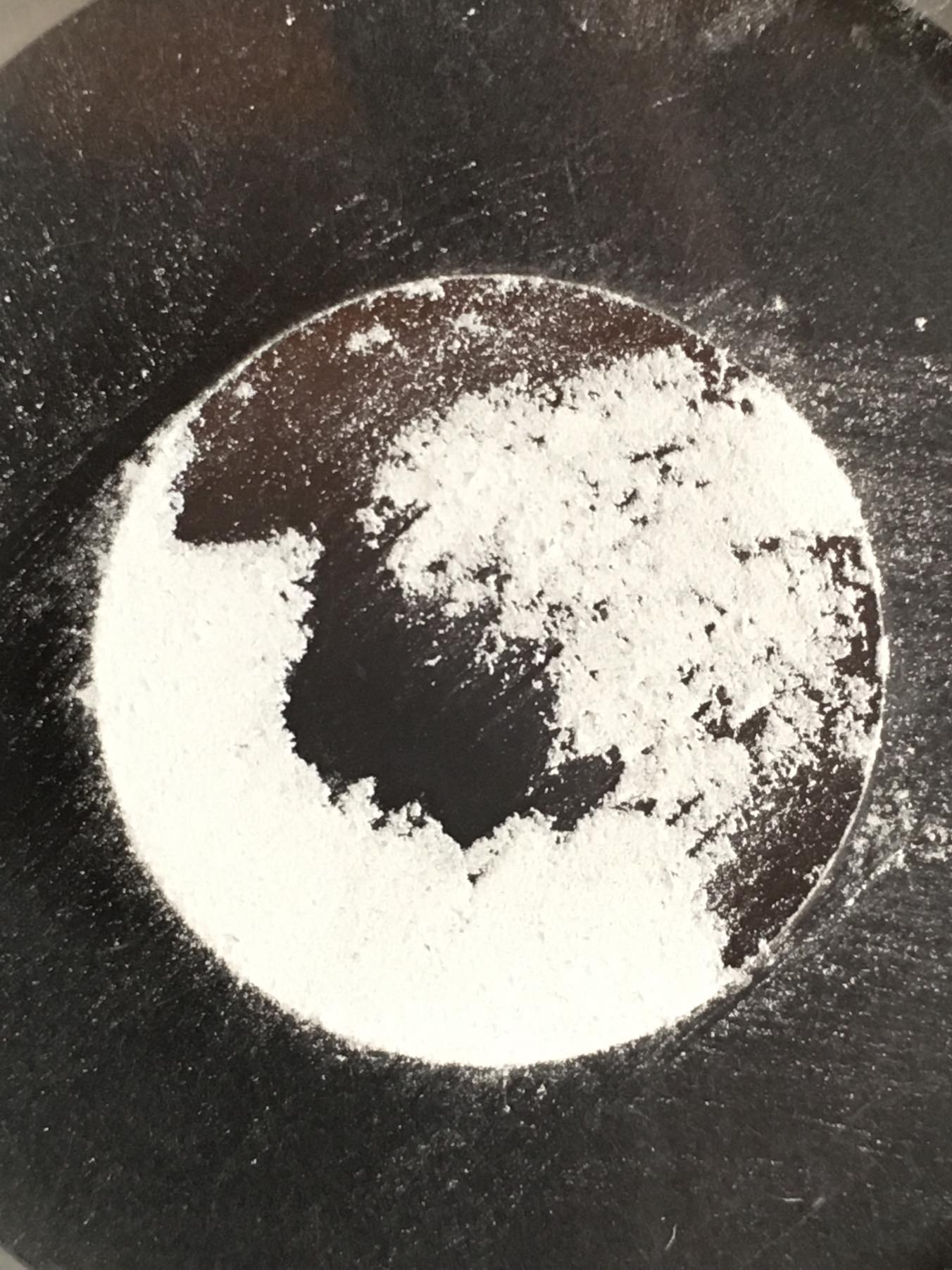

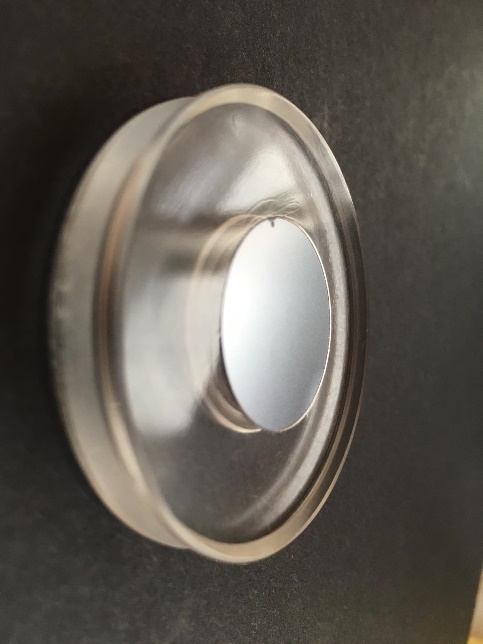


**Supplementary Figure 1**: Silicon pod (depth 0.1 mm and diameter 24.0 mm) with a 7.5 mg of sample.

# **Cocaine**

The HighScore Plus is a commonly used software for either processing of diffraction patterns or phase identification of unknown samples. It uses automatic in-build procedures to work with collected data and it contains tools for comparing the measured data with diffraction patterns present in a database (PDF files). The pattern searching within the database is a standardised and quite simple to use procedure. PDF4+ database containing more than 410 000 diffraction patterns is commercially available. Unlike NPSs, this database contains diffraction patterns of heroin and cocaine. Such big amount of patterns in this database made us wonder, if it would be possible to use this database for identification of the street samples of heroin and cocaine. Results were quite impressive; as we were able to identify both cocaine and heroin in the street mixtures (see non-modified search data in the Fig. **S1**, **S2** and **S6**). However, we were unable to assign the cutting agents, as this database mostly does not contain their patterns. The proper way for XRPD would be the identification of the main compound first and then assigning all of the other signals in the pattern to other compounds. The appropriate database containing not only psychoactive substances but also cutting agents would mostly solve this issue as remaining signals may thus be assigned. Currently, such database focused on psychoactive substances and cutting agents does not exist, but we believe that in the future, when this technique starts to appear in the forensics, appropriate database will become available.


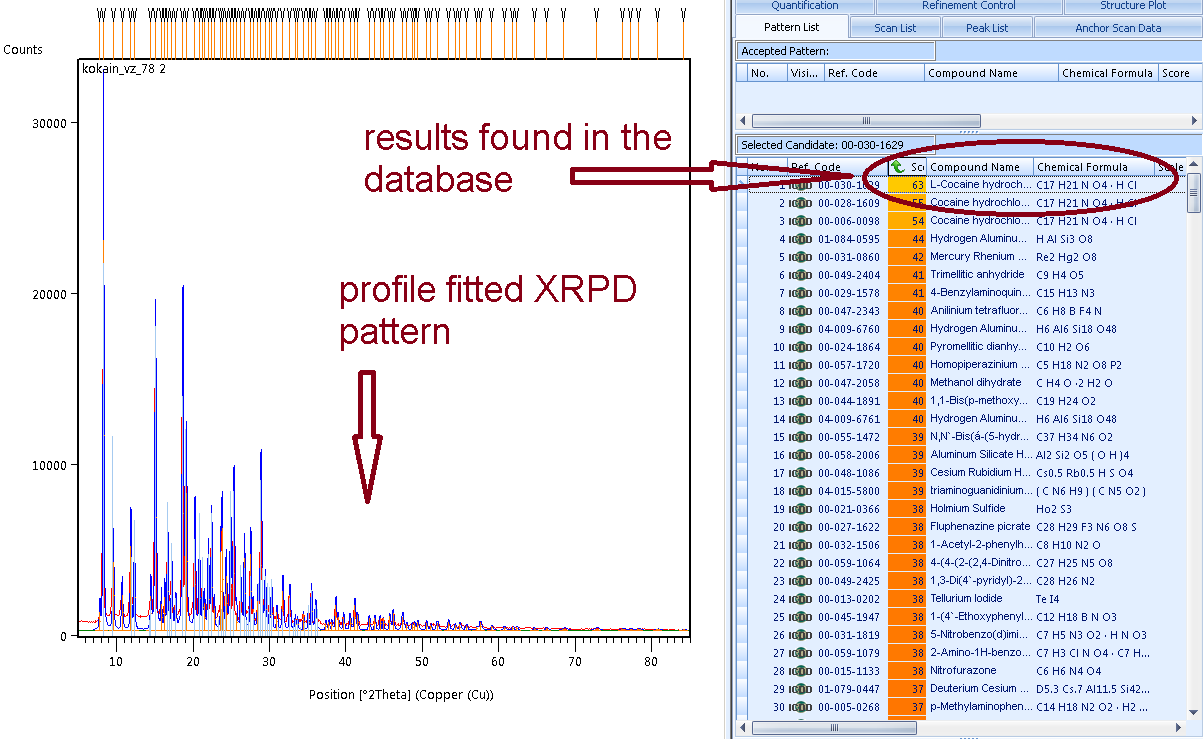


**Supplementary Figure 2**: Printscreen from a HighScore Plus software using the inner PDF4+ database for the identification of street cocaine


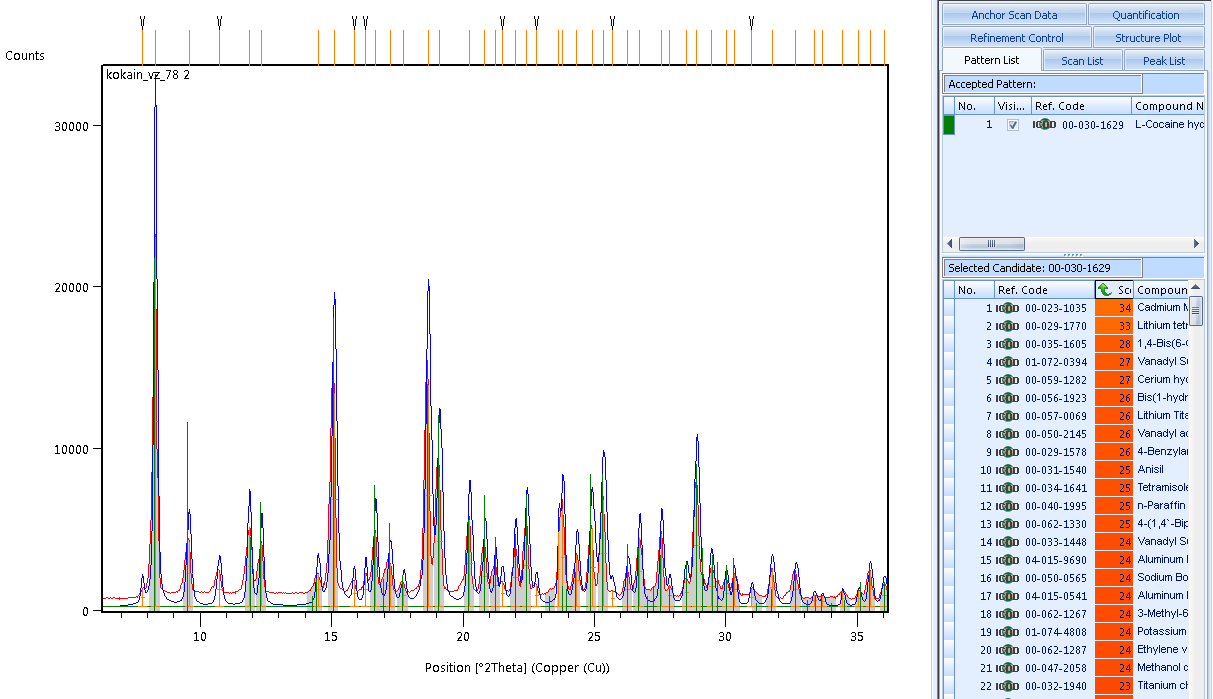


**Supplementary Figure 3**: Printscreen from a HighScore Plus software using the inner PDF4+ database for the identification of street cocaine (Detail)


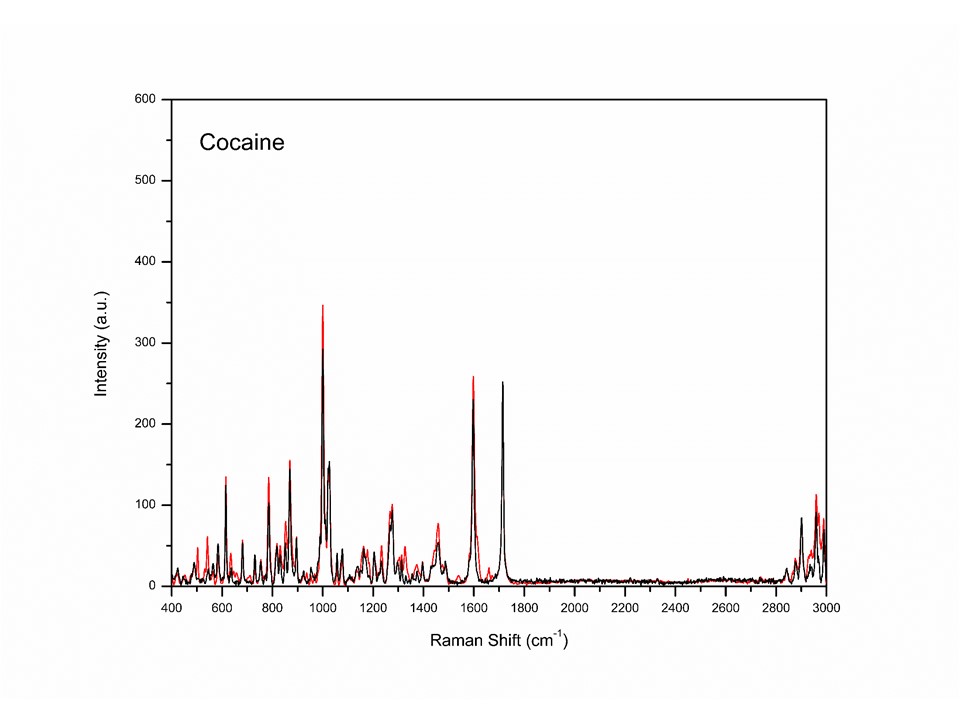


**Supplementary Figure 4**: Raman spectra using 780 nm laser of the Cocaine. Red line marks the seized samples and black marks the standards.


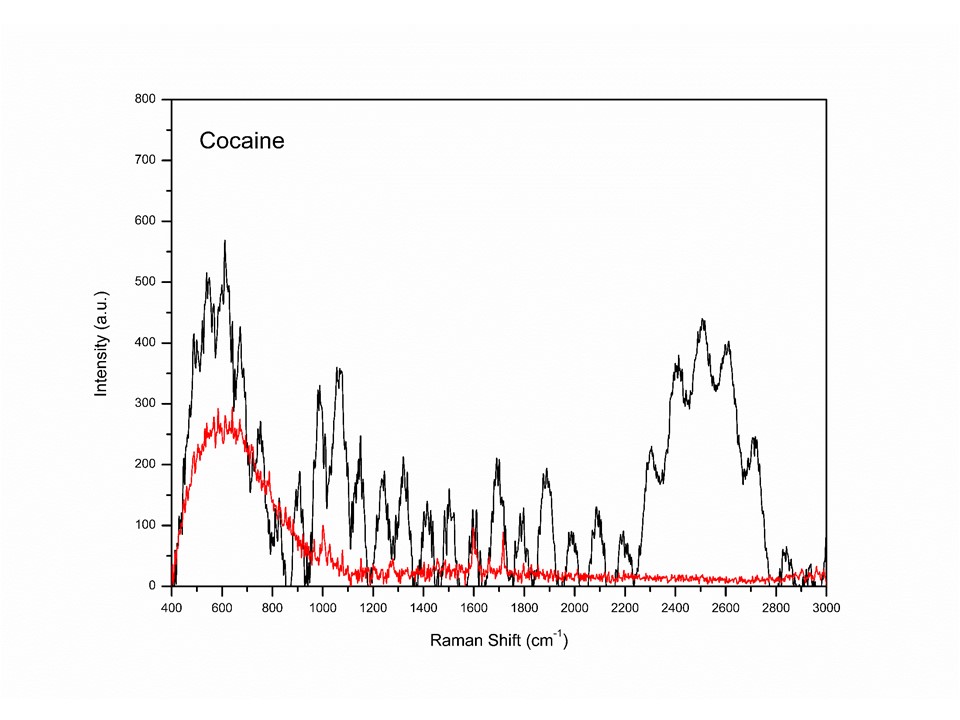


**Supplementary Figure 5**: Raman spectra using 532 nm laser of the Cocaine. Red line marks the seized samples and black marks the standards.


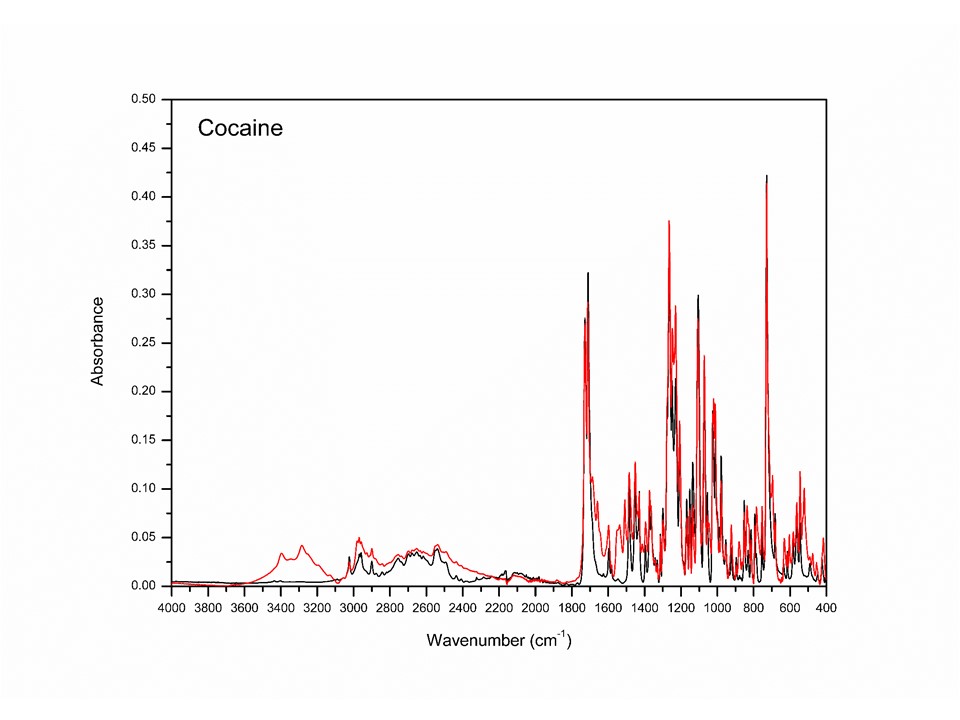


**Supplementary Figure 6**: IR spectra of the Cocaine. Red line marks the seized samples and black marks the standards.

# Heroin


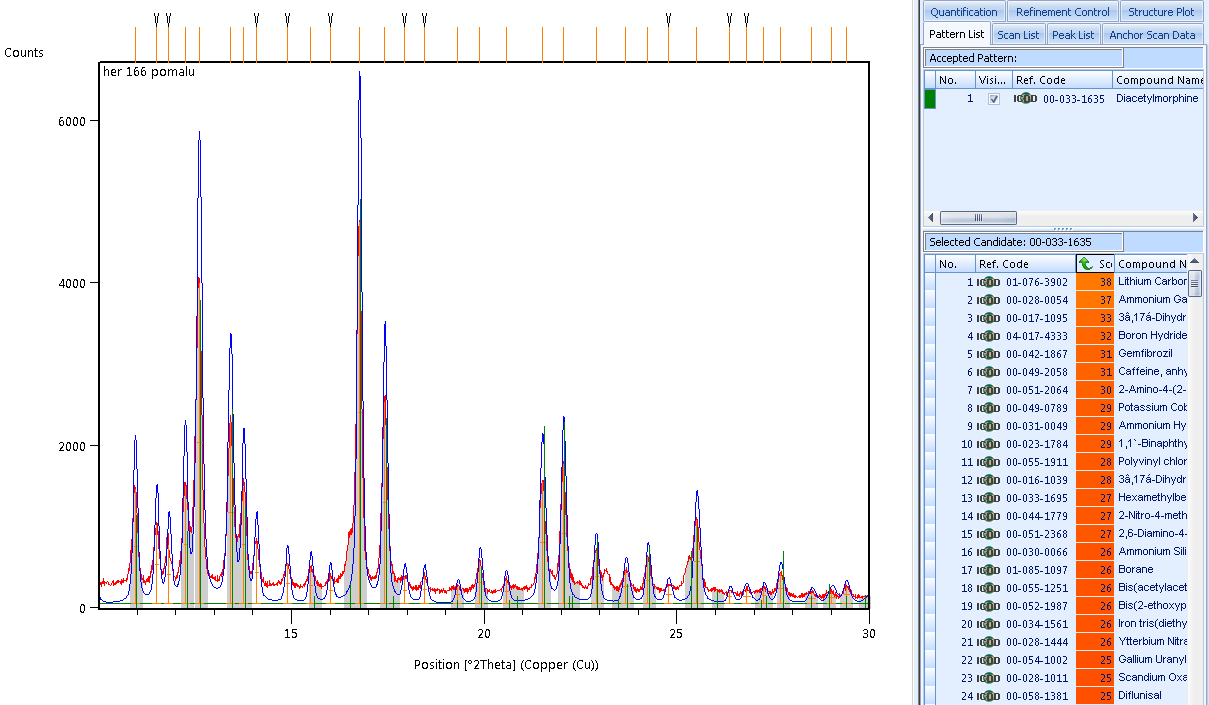


**Supplementary Figure 7**: Printscreen from a HighScore Plus software using the inner PDF4+ database for the identification of street heroin (detail)


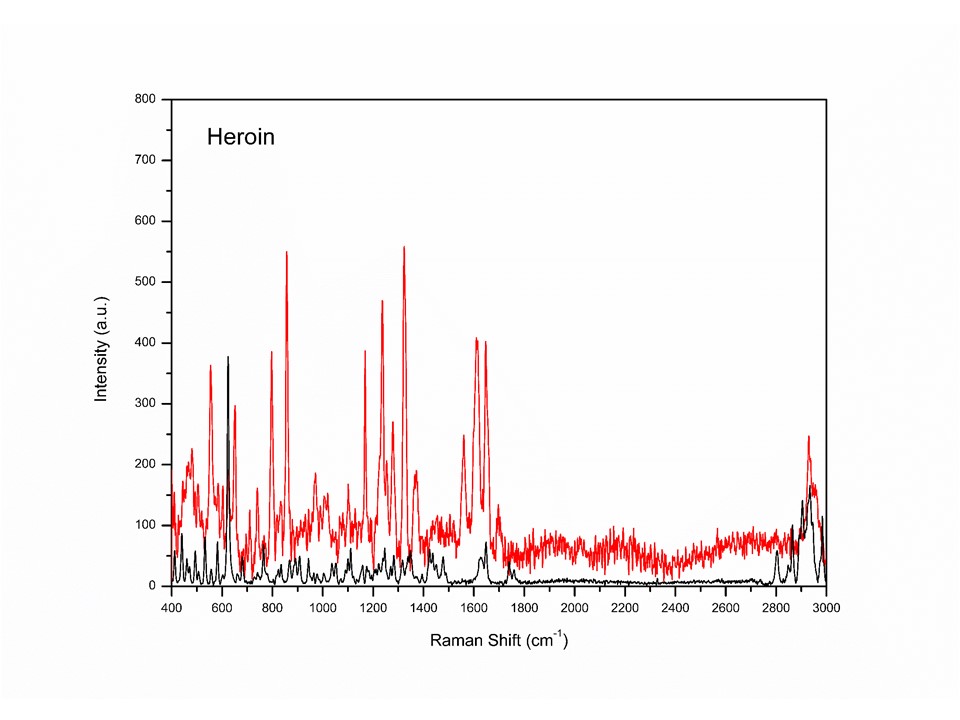


Supplementary Figure 8: Raman spectra using 780 nm laser of the Heroin. Red line marks the seized samples and black marks the standards.


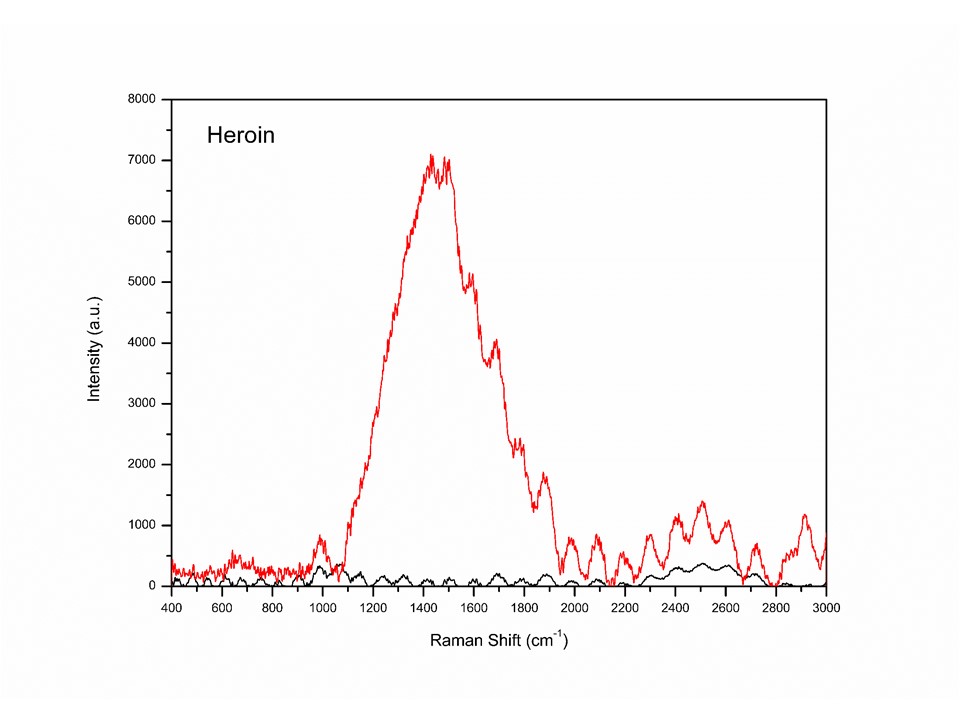


**Supplementary Figure 9**: Raman spectra using 532 nm laser of the Heroin. Red line marks the seized samples and black marks the standards.


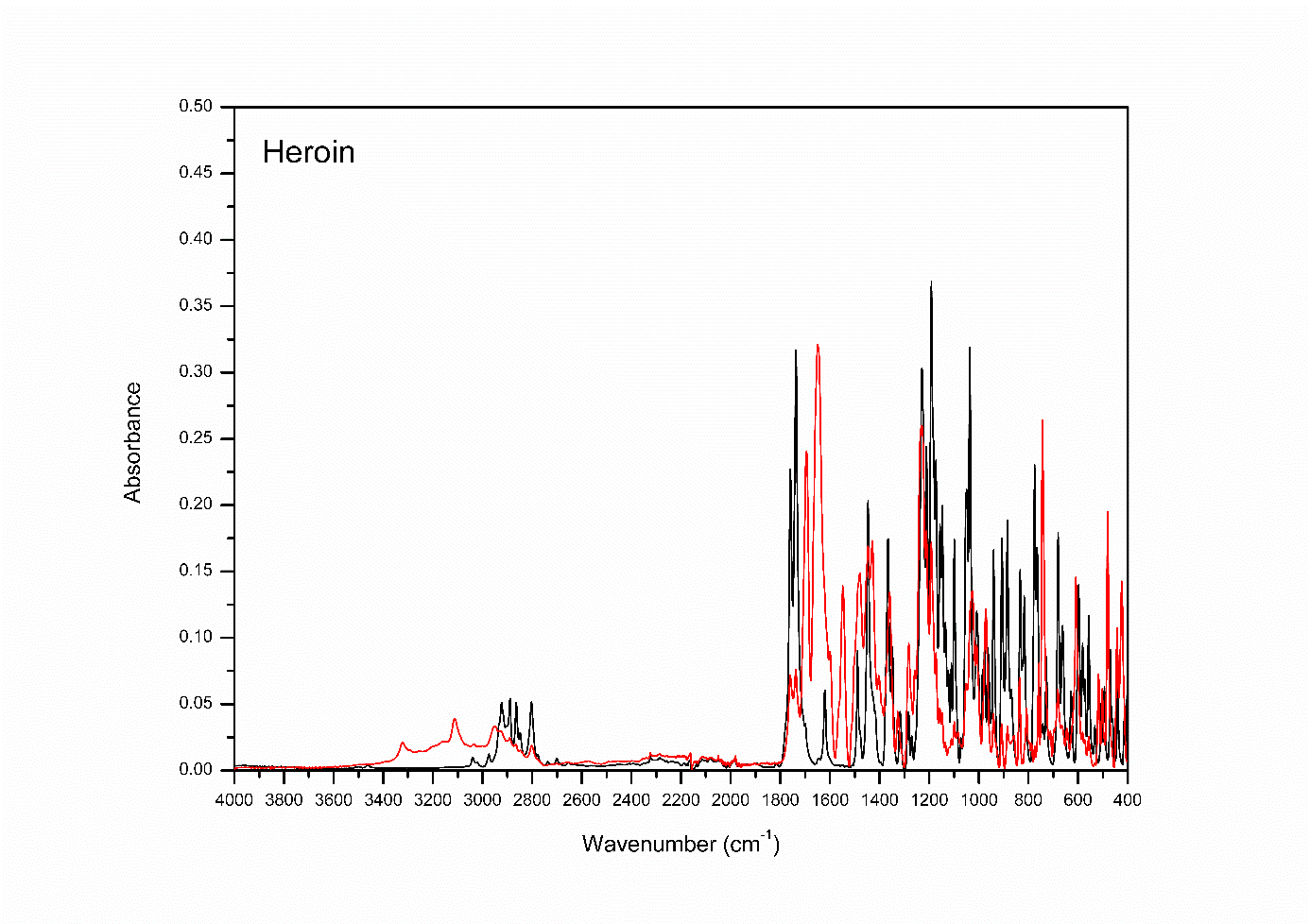


**Supplementary Figure 10**: IR spectra of the Heroin. Red line marks the seized samples and black marks the standards.

# **Mephedrone**


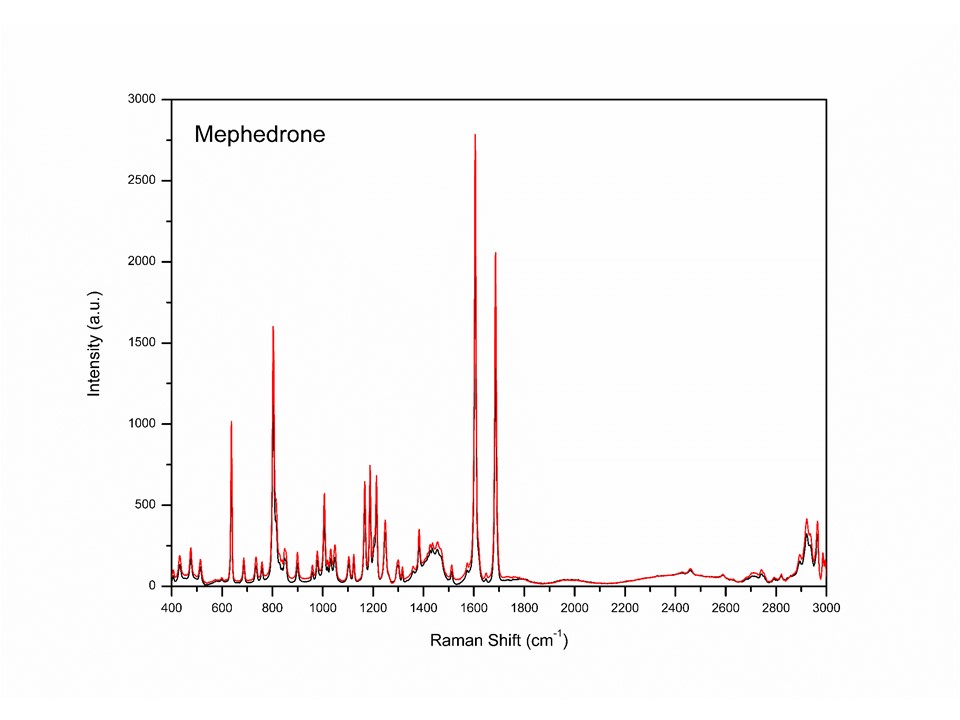


**Supplementary Figure 11**: Raman spectra using 780 nm laser of the Mephedrone. Red line marks the seized samples and black marks the standards.


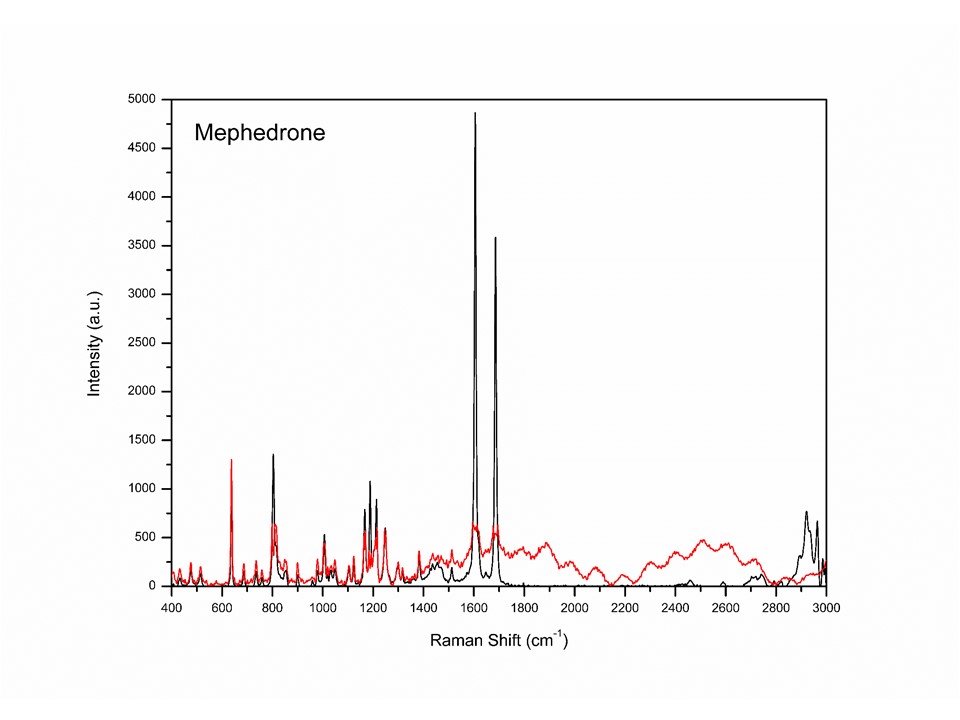


**Supplementary Figure 12**: Raman spectra using 532 nm laser of the Mephedrone. Red line marks the seized samples and black marks the standards.


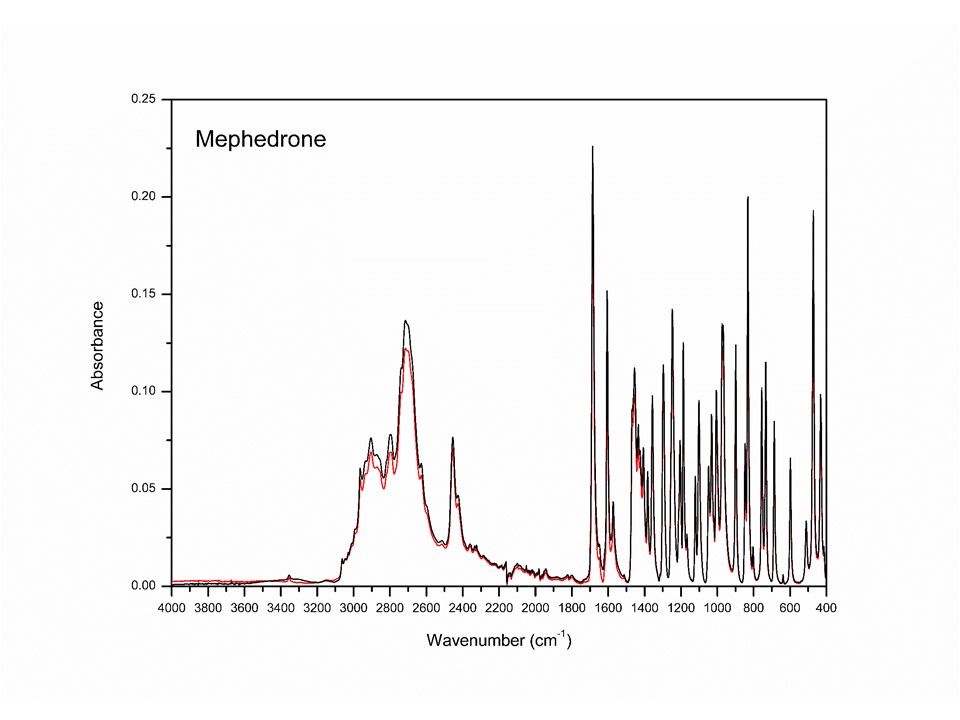


**Supplementary Figure 13**: IR spectra of the Mephedrone. Red line marks the seized samples and black marks the standards.

# **Ephylone**


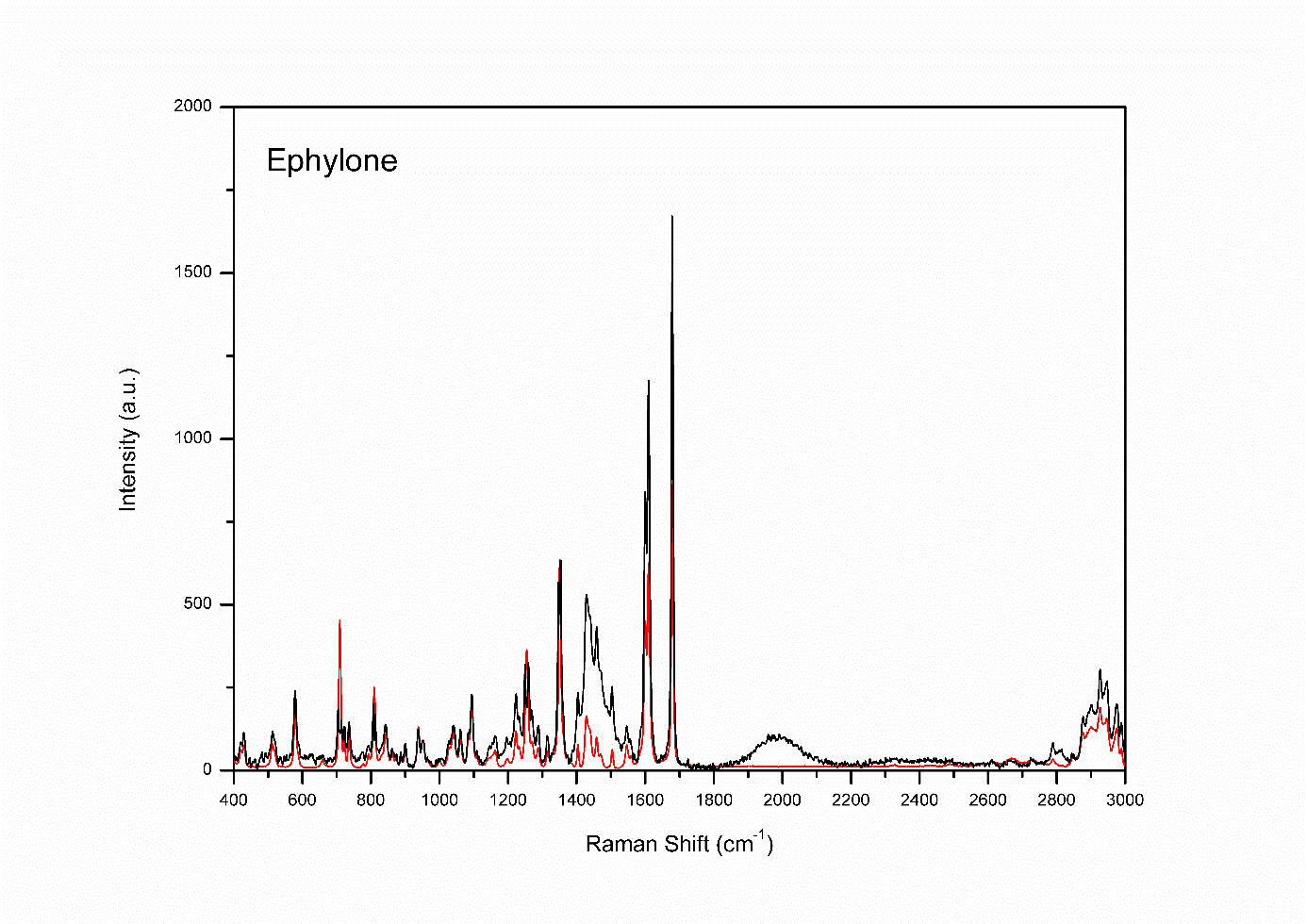


**Supplementary Figure 14**: Raman spectra using 780 nm laser of the Ephylone. Red line marks the seized samples and black marks the standards.


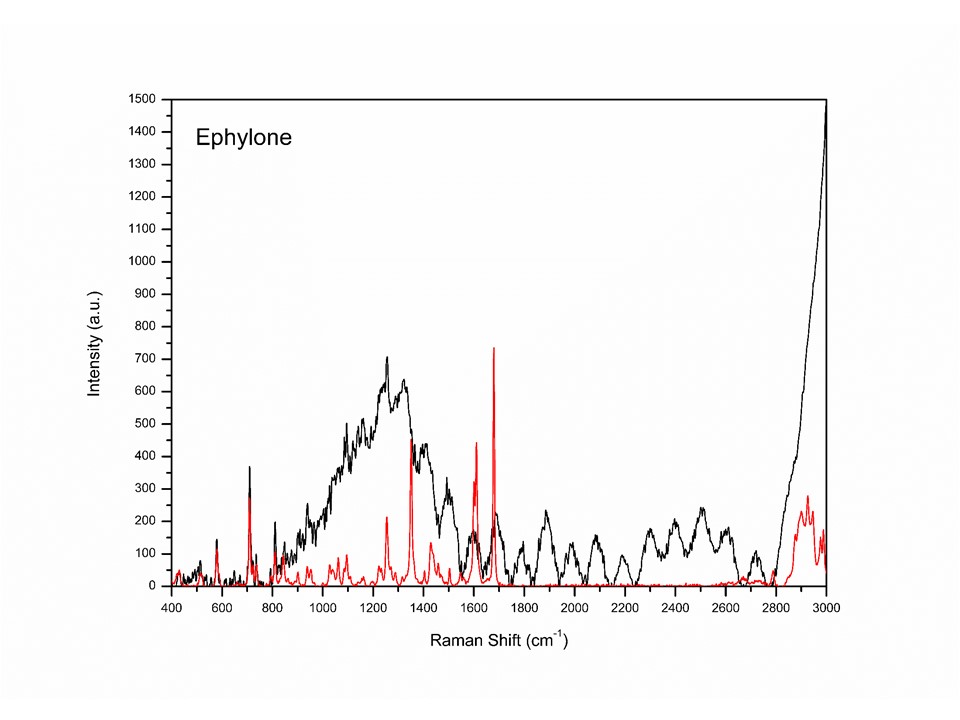


**Supplementary Figure 15**: Raman spectra using 532 nm laser of the Ephylone. Red line marks the seized samples and black marks the standards.


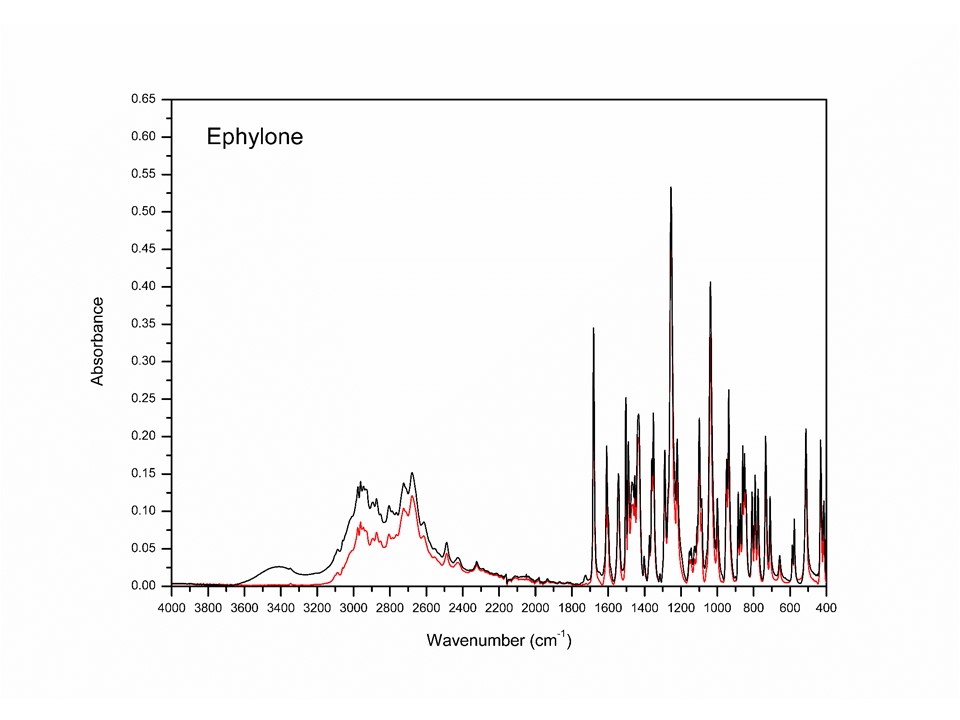


**Supplementary Figure 16**: Raman spectra using 780 nm laser of the Ephylone. Red line marks the seized samples and black marks the standards.

# **Butylone**


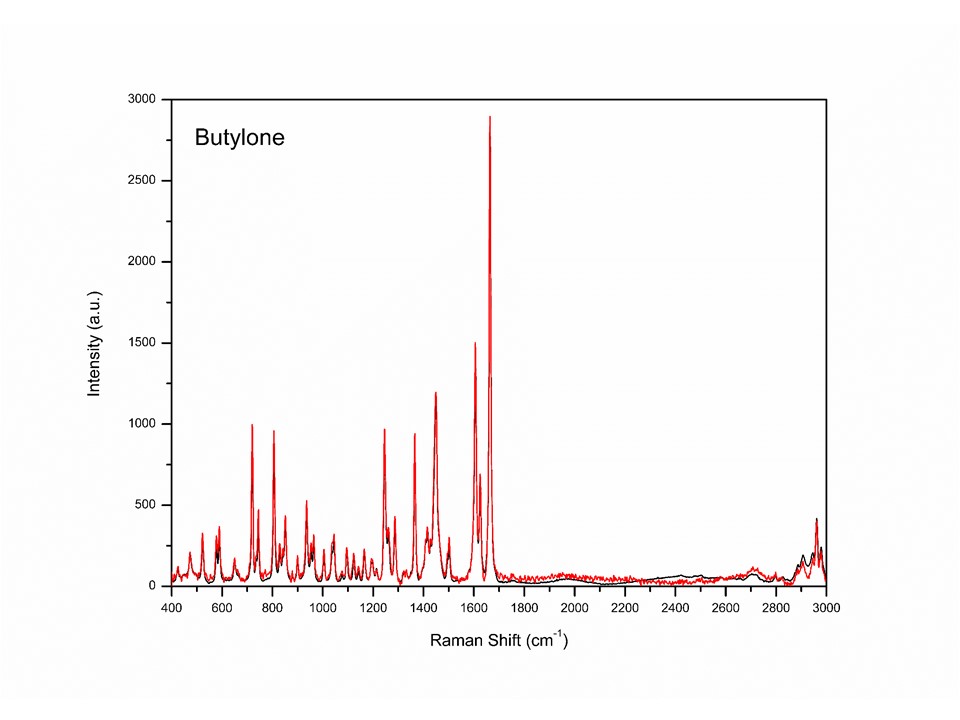


**Supplementary Figure 17**: Raman spectra using 780 nm laser of the Butylone. Red line marks the seized samples and black marks the standards.


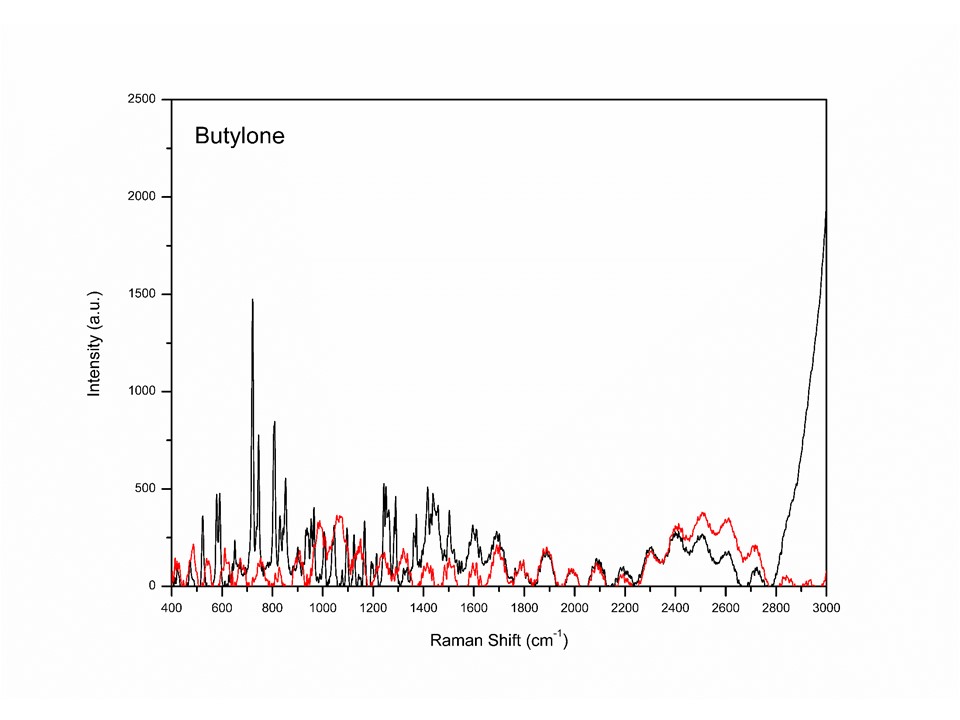


**Supplementary Figure 18**: Raman spectra using 532 nm laser of the Butylone. Red line marks the seized samples and black marks the standards.


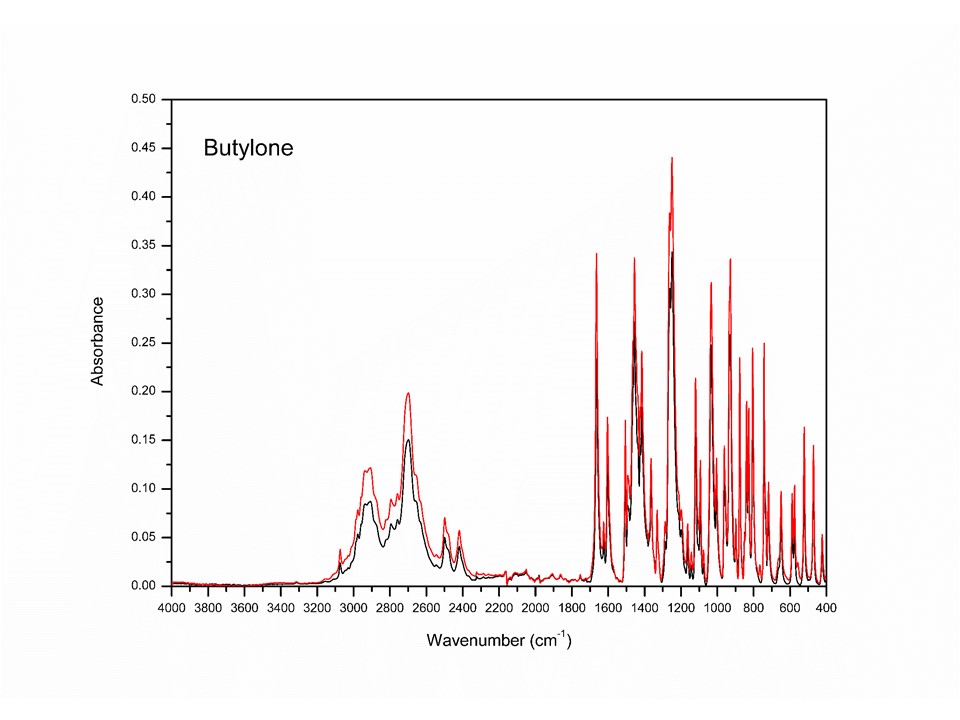


**Supplementary Figure 19**: IR spectra of the Butylone. Red line marks the seized samples and black marks the standards.

# **JWH-073**


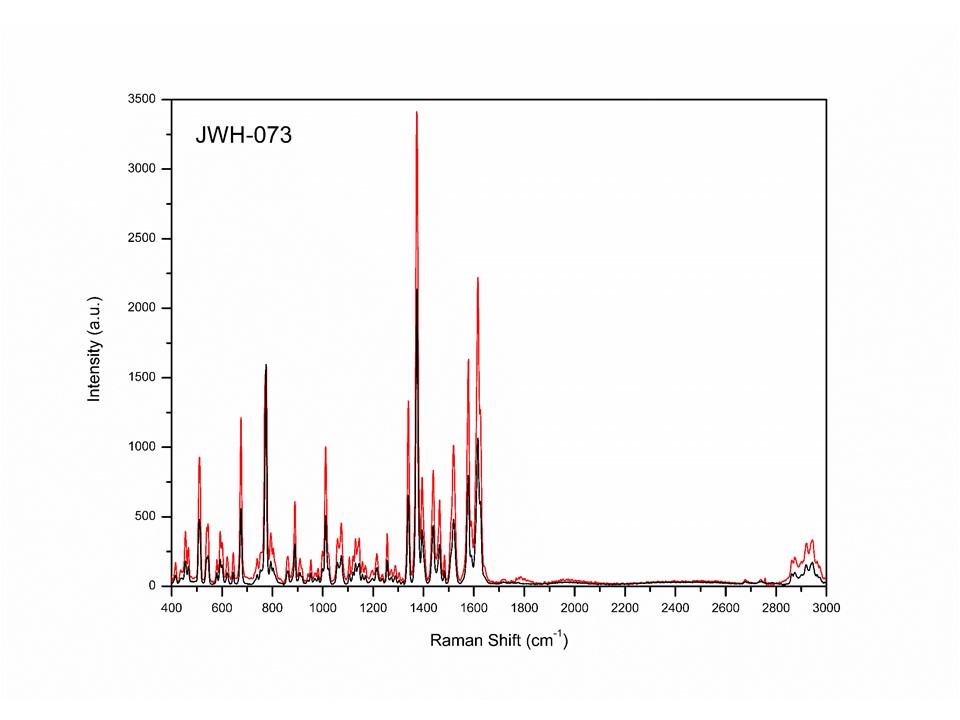


**Supplementary Figure 20**: Raman spectra using 780 nm laser of the JWH-073. Red line marks the seized samples and black marks the standards.


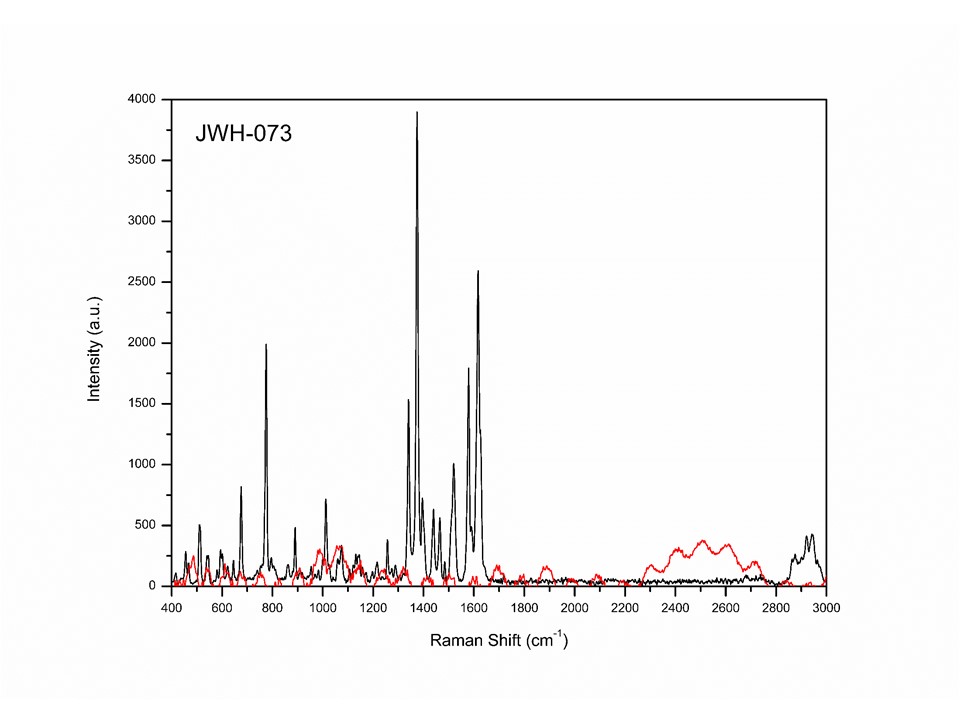


**Supplementary Figure 21**: Raman spectra using 532 nm laser of the JWH-073. Red line marks the seized samples and black marks the standards.


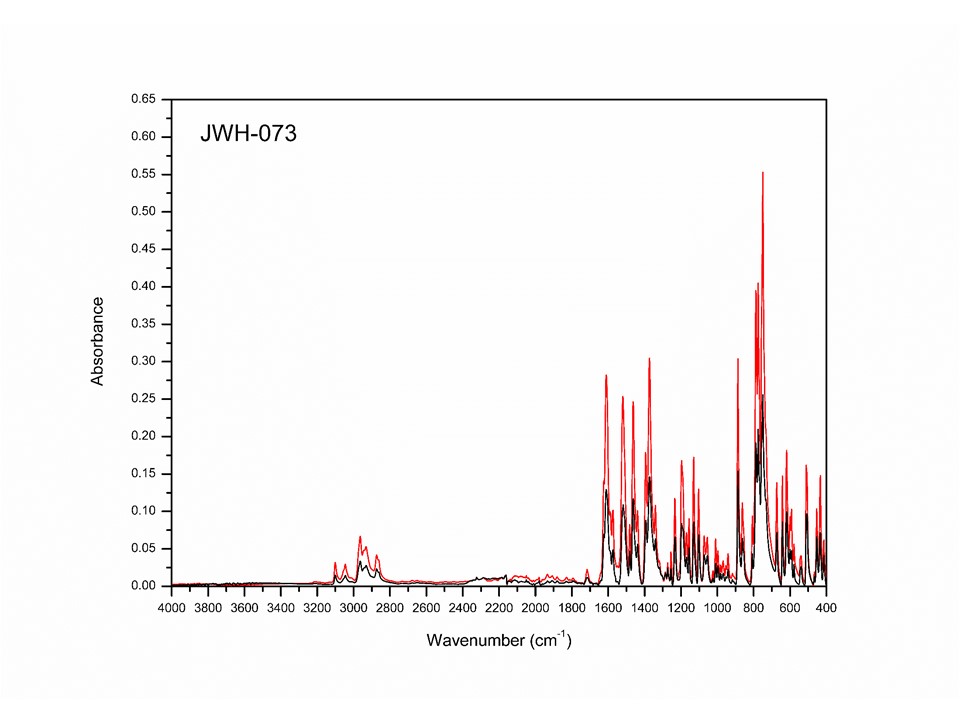


**Supplementary Figure 22**: IR spectra of the JWH-073. Red line marks the seized samples and black marks the standards.

# **Naphyrone**


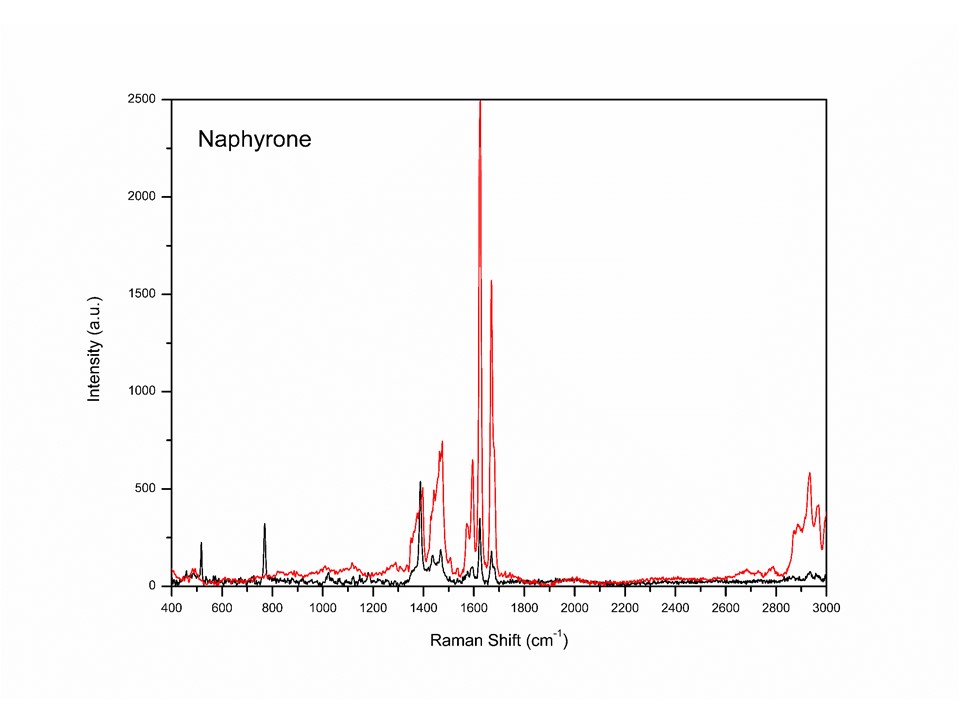


**Supplementary Figure 23**: Raman spectra using 780 nm laser of the Naphyrone. Red line marks the seized samples and black marks the standards.


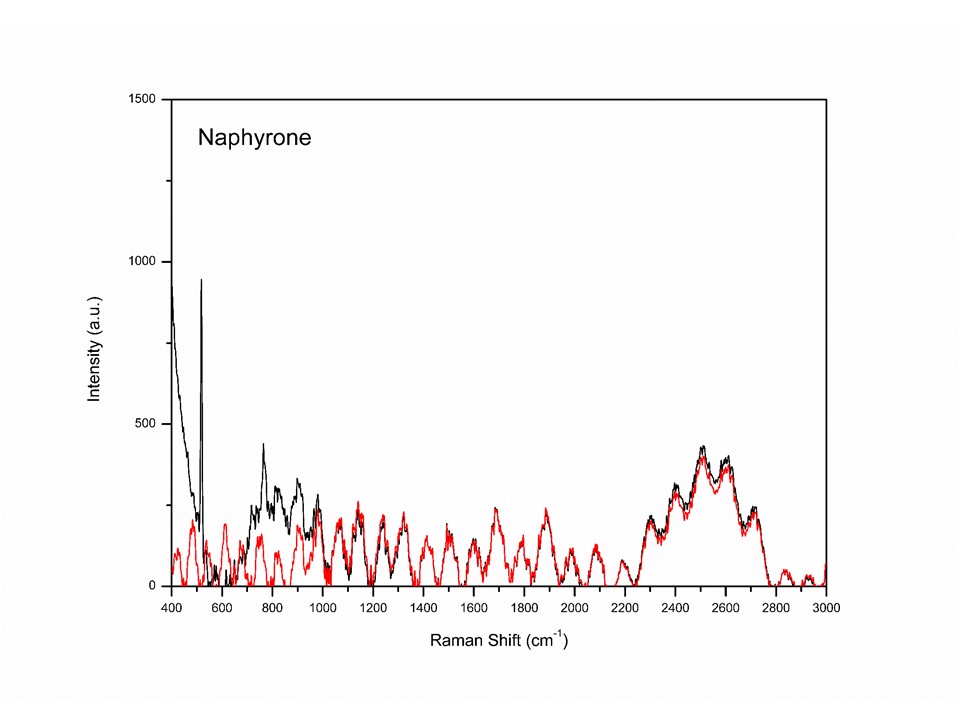


**Supplementary Figure 24**: Raman spectra using 532 nm laser of the Naphyrone. Red line marks the seized samples and black marks the standards.


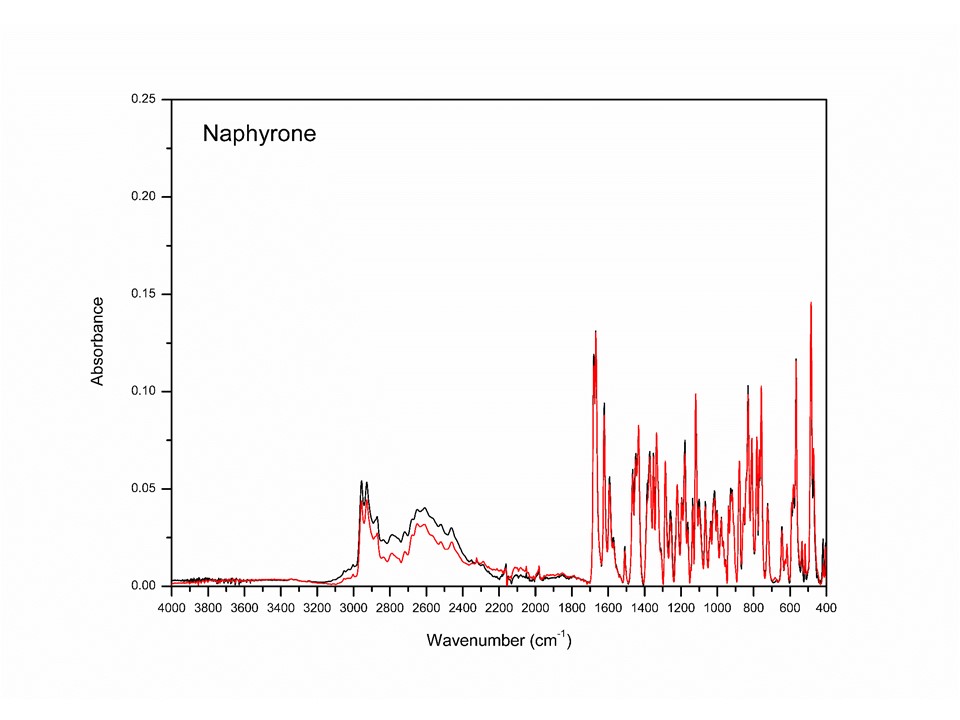


**Supplementary Figure 25**: IR spectra of the Naphyrone. Red line marks the seized samples and black marks the standards.
